# BRCA1 heterozygosity promotes DNA damage-induced senescence in a sex-specific manner following repeated mild traumatic brain injury

**DOI:** 10.3389/fnins.2023.1225226

**Published:** 2023-08-10

**Authors:** Emily Leung, Daria Taskina, Nicole Schwab, Lili-Naz Hazrati

**Affiliations:** ^1^Department of Laboratory Medicine and Pathobiology, University of Toronto, Toronto, ON, Canada; ^2^Neurosciences and Mental Health, The Hospital for Sick Children, Toronto, ON, Canada

**Keywords:** mild traumatic brain injury, BRCA1, DNA damage, DNA repair, cellular senescence, sex difference

## Abstract

Emerging evidence suggests cellular senescence, as a consequence of excess DNA damage and deficient repair, to be a driver of brain dysfunction following repeated mild traumatic brain injury (rmTBI). This study aimed to further investigate the role of deficient DNA repair, specifically BRCA1-related repair, on DNA damage-induced senescence. BRCA1, a repair protein involved in maintaining genomic integrity with multiple roles in the central nervous system, was previously reported to be significantly downregulated in post-mortem brains with a history of rmTBI. Here we examined the effects of impaired BRCA1-related repair on DNA damage-induced senescence and outcomes 1-week post-rmTBI using mice with a heterozygous knockout for BRCA1 in a sex-segregated manner. Altered BRCA1 repair with rmTBI resulted in altered anxiety-related behaviours in males and females using elevated zero maze and contextual fear conditioning. Evaluating molecular markers associated with DNA damage signalling and senescence-related pathways revealed sex-specific differences attributed to BRCA1, where females exhibited elevated DNA damage, impaired DNA damage signalling, and dampened senescence onset compared to males. Overall, the results from this study highlight sex-specific consequences of aberrant DNA repair on outcomes post-injury, and further support a need to develop sex-specific treatments following rmTBI.

## Introduction

Mild traumatic brain injury (mTBI) is a growing public health issue highly associated with disability and an increased risk for neurodegeneration later in life (Gardner and Yaffe, 2015). Impacting millions of individuals every year, mTBI is accompanied by alterations in wakefulness, mood-related symptoms, and reduced cognitive performance (Dean and Sterr, 2013; Pavlovic et al., 2019), where repeated, compared to single, injury can promote more severe deficits and increased prevalence of cognitive impairment due to cumulative effects (Guskiewicz et al., 2005). Most individuals recover within a few weeks to months, however others may experience persisting post-concussive symptoms long-term (Hiploylee et al., 2017; McInnes et al., 2017). In addition to primary injury, several mechanisms associated with secondary injury, such as oxidative stress and DNA damage, have been established to promote long-term consequences and neurodegeneration (Bramlett and Dietrich, 2015; Schwab et al., 2019; Davis and Vemuganti, 2021). These mechanisms promote the production of both single and double-stranded breaks, increasing the burden of genotoxic stress post-injury. As a response, the DNA damage response (DDR) is activated, recruiting DNA damage sensing and repair proteins to resolve DNA lesions. However, with persistent activation and insufficient repair, excess DNA damage can induce the onset of cellular senescence (Sedelnikova et al., 2004; Jurk et al., 2012), a state of permanent cell-cycle arrest characterized by aberrant structural and functional changes, accompanied by a pro-inflammatory senescence associated secretory phenotype (SASP) that leads to cellular and tissue dysfunction over time (Coppé et al., 2010; Davalos et al., 2010). Accordingly, evidence of cellular senescence and brain dysfunction have been reported in mTBI (Schwab et al., 2019, 2022) and neurodegeneration (Salminen et al., 2011; Baker and Petersen, 2018).

Previously, we demonstrated DNA damage-induced senescence as a pathophysiological mechanism driving brain dysfunction in humans and rodent models post-mTBI (Schwab et al., 2019, 2021), where an upregulation in genes associated with DNA damage and senescence were observed, in addition to a downregulation in DNA repair genes. Among these DNA repair genes, breast cancer type I (BRCA1) was seen to be significantly downregulated in post-mortem brains of professional athletes with a history of repeated mTBI (rmTBI) (Schwab et al., 2019). As a key protein in the DDR, BRCA1 is involved in multiple repair pathways for both single- and double-stranded breaks, including base excision repair (Saha et al., 2010), homologous recombination (Liu and Lu, 2020), and non-homologous end joining (Baldeyron et al., 2002; Jiang et al., 2013). With more specificity towards the central nervous system, BRCA1 also functions in the brain throughout neurodevelopment (Pao et al., 2014) and adulthood, with its dysregulation reported in multiple neurodegenerative diseases (Noristani et al., 2015; Suberbielle et al., 2015; Kurihara et al., 2020). Furthermore, BRCA1 loss has also been implicated in cellular senescence in several tissue types (Tu et al., 2013; Sedic et al., 2015; Scott et al., 2017), however its involvement in brain senescence remains to be investigated.

In relation to mTBI, sex-specific differences in response, recovery and outcomes are well established, where women often report worse outcomes, more severe symptoms and longer recovery times (Bazarian et al., 2010; Gupte et al., 2019). However despite these discrepancies, women are often underrepresented in TBI research (Merritt et al., 2019). Sex differences in the context of cellular senescence (Ng and Hazrati, 2022) and neurodegeneration (Hanamsagar and Bilbo, 2016; Ullah et al., 2019) are also well documented. As a precursor and prominent feature of senescence and neurodegeneration, emerging evidence also highlights sex differences in the DNA damage response (Broestl and Rubin, 2021) and DNA repair capacity with aging (Rall-Scharpf et al., 2021). Whether BRCA1 plays an important role in sex-specific outcomes following rmTBI is currently unknown.

In this study, mice with a heterozygous knockout for BRCA1 in conjunction with a murine model of rmTBI was used to investigate the role of DNA repair deficiency in DNA damage-induced senescence and sex-specific outcomes post-injury. Our results support elevated DNA damage, followed by divergent molecular and behavioural outcomes 1-week post-injury due to BRCA1 heterozygous knockout in a sex-specific manner.

## Methods

### Animals

Adult (8–10 weeks old) male and female mice (Brca1^tm1Cxd^/Nci, strain# 01XC4, Frederick National Laboratory NCI Mice Repository) were used in this study, producing both wildtype (WT) and BRCA1 heterozygous knockout (HET) agouti offspring (Shen et al., 1998). Tail clippings were genotyped by Transnetyx Genotyping services. Mice were housed in a 12 h light–dark cycle under standard laboratory conditions, with access to food and water *ad libitum*. Separate cohorts of mice were used for behavioural testing, tissue analysis, and histology experiments. All experiments were approved by the Centre of Phenogenomics Animal Care Committee.

### Repeated mild traumatic brain injury

Mice received mTBI using a closed skull controlled cortical impact injury model. To achieve repeated injury, mice received three consecutive impacts 24 h apart. On day 1 and 3 of surgeries, mice were given sustained release buprenorphine (1.2 mg/kg) as an analgesic. Mice were anesthetized with isoflurane at 4% and maintained under the surgical plane at 2%. Additional subcutaneous injections of 0.75 mL lactated ringers with 5% dextrose, and 0.1 mL bupivacaine (2.5 mg/mL) and xylocaine (1.0 mg/mL) in equal parts were also given. Scalp hair removal was completed, followed by a midline incision to expose the closed skull. Once the skull was levelled, an impact was given using the Leica Impact One Stereotaxic Impactor (Leica Biosystems) with a 5 mm metal tip positioned 2.5 mm right of Bregma (speed: 2 m/s, depth: 1,200 um, dwell time: 200 ms). Sham mice underwent the same procedure but without impact. Following impact, the incision was sutured closed and mice were placed in a recovery cage before being returned to their home cage with food and nesting material. Righting reflex was also recorded, analyzed using a repeated measures two-way ANOVA, and visualized using GraphPad Prism 8.0.1.

### Behavioural testing

At 1-week post-injury, mice underwent behavioural testing that included the elevated zero maze and contextual fear conditioning. Prior to testing, mice were placed in an anteroom, undisturbed, for 30 min. Mice also underwent a phenotype assessment prior to behavioural tests, measuring weight, length, locomotor activity and reflexes to assess any baseline differences (*n* = 7–9 per group). Behavioural testing was conducted over three consecutive days, where elevated zero maze took place on the first day, followed by contextual fear conditioning on the second day, and contextual fear testing on the third day. Data collected for these behavioural tests took place over several months and involved analysis of multiple cohorts of mice.

*Elevated zero maze (EZM)* assessed anxiety-related behaviours. Mice were placed in the arena (diameter: 60 cm, height: 70 cm, arm width: 7 cm, Med Associates Inc.) for a duration of 5 min (300 s), starting in an open arm facing the entrance of a closed arm (*n* = 10–15 per group). Metrics recorded included distance travelled, mean velocity, and time spent in the open/closed arms using an overhead camera and EthoVision XT software (Noldus Information Technology).

*Contextual fear conditioning (CFC)* assessed associative learning and memory. Mice were placed in fear conditioning chambers (length: 60 cm, width: 71 cm, height: 32 cm, Med Associates Inc.) for a total duration of 5 min (300 s) (*n* = 6–10 per group). Foot shocks of 0.75 A as the aversive unconditioned stimulus were administered using A/S Aversive Stimulator boxes (Med Associates Inc.). 70% isopropyl alcohol was used as the olfactory contextual cue. The conditioning protocol consisted of a 120 s baseline, three 2 s shocks with a 58 s interval between each shock, and a 58 s post-shock period. Mice were then returned to the arena 24 h following conditioning for a duration of 5 min (300 s), receiving no shocks. Metrics recorded included average motion index and time spent freezing using the NIR Video Fear Conditioning System (Med Associates Inc.).

Raw data was analyzed with a two-way ANOVA for injury and phenotype, followed by unpaired *t*-tests for pairwise comparisons. For pairwise comparisons, experimental groups were compared to WT sham, with each rmTBI group compared to its respective sham (i.e., WT rmTBI to WT sham, and HET rmTBI to HET sham) separated by sex. Statistical significance was denoted as *p* < 0.05. For data that did not follow an approximately normal distribution, a log transformation was performed before statistical analysis so that the requirements for a two-way ANOVA could be met. Results were visualized using GraphPad Prism 8.0.1.

### Animal sacrifice

Mice were sacrificed 1-week post-injury for tissue analysis and histology via transcardial perfusion under anesthesia. Ketamine (150 mg/kg; 100 mg/mL) and xylazine (10 mg/kg; 20 mg/mL) diluted in saline was used for anesthetization with an injected volume equating to 0.1 mL/10 g body weight plus 30% (i.e., body weight × 1.3). Mice were perfused with PBS and heparin followed by brain dissection and flash freezing in 2-methyl butane placed in liquid nitrogen for tissue analysis. Mice sacrificed for histology were perfused first with PBS and heparin before a second perfusion with 4% paraformaldehyde, followed by brain dissection and post-fixation in 4% paraformaldehyde. Mice undergoing behavioural testing 1-week post-injury were sacrificed using carbon dioxide on the last day of testing.

### Real time PCR (qPCR)

RNA isolation was performed on cortical tissue (*n* = 3–6 per group) from the right hemisphere (i.e., ipsilateral to impact) using TRIzol reagent (Invitrogen; 1 mL/50 mg tissue) and chloroform (Sigma-Aldrich; 200 uL/1 mL TRIzol). Purity and RNA concentration was measured using the Nanodrop One UV–Vis Spectrophotometer (Thermofisher) prior to reverse transcription of 2.5 ug RNA with SuperScript IV VILO Master Mix (Invitrogen 11,756,050). cDNA was combined with 500 nM forward and reverse primers (Sigma-Aldrich) and SYBR Green Master Mix (Applied Biosystems 4,472,908), and run in triplicates (50 cycles, annealing temperature: 61°C) for qPCR using QuantaStudio 3 instrument (Applied Biosystems). All primer sequences were taken from literature, with one designed using OligoArchitect™ Primer and Probe Design online tool (Sigma-Aldrich). A complete list of target genes and sequences can be found in Supplementary Table S1.

Raw data was analyzed using the 2^−ΔΔCt^ method, averaging cycle values (Ct) and normalizing to GAPDH as the housekeeping gene. ΔΔCt values were calculated relative to an internal sham control for each experimental group. Results from male and female mice were analyzed separately using a two-way ANOVA for injury and genotype, with statistical significance denoted as *p* < 0.05. Effect size (η^2^) was calculated and unpaired *t*-tests were performed subsequently comparing experimental groups to WT sham, and each rmTBI group to its respective sham. Results were represented on a log_2_ fold change scale and visualized using GraphPad Prism 8.0.1.

### Western blots

Protein lysates were prepared with cortical tissue (*n* = 4–8 per group) from the right hemisphere (i.e., ipsilateral to impact) using phosphate-buffered saline (PBS; 1 mL/50 mg tissue) containing proteinase and phosphatase inhibitor cocktail (1:100, Thermofisher). Protein concentration was determined via Bradford Assay using Bradford reagent (Sigma-Aldrich B6916) and BSA Protein Standard (Bio-Rad 500–0007). 50 ug of protein was loaded per sample on 10% polyacrylamide gels prepared using TGX™ Fast Cast™ Acrylamide kit (Bio-Rad 1,610,173), and run at constant voltage alongside a 75 kDa protein ladder (FroggaBio PM008-0500). Gels were transferred onto nitrocellulose membranes (Bio-Rad 1,620,112) and blocked in 2% bovine serum albumin (BSA) (Sigma Aldrich) in tris-buffered saline with 0.05% tween (TBST) for 2 h at room temperature. Membranes were incubated overnight in primary antibodies prepared in 1% BSA in TBST. Primary antibodies included BRCA1 (1:1000, Santa Cruz SC-135732), γH2AX (Ser139) (1:2,000, Cell Signalling 2,577), pATM (Ser1981) (1:2,000, Millipore MAB3806-C), DNA2 (1:2,000, Thermofisher PA5-68167), p16 (1:2,000, Santa Cruz SC-1661), p21 (1:2,000, BD Pharmingen 556,430), and GAPDH (1:2,000, Cell Signalling 14C10). Membranes were washed three times in PBS with 0.05% tween (PBST) for 5 min, followed by incubation in anti-mouse (1:10,000, Sigma-Aldrich A9044) and anti-rabbit (1:20,000, Sigma-Aldrich A0545) horse radish peroxidase (HRP)-conjugated secondary antibodies prepared in 1% BSA in TBST for 2 h at room temperature. Membranes were washed once for 15 min with PBST, followed by two 15 min washes in PBS. Enhanced chemiluminescence substrate (Bio-Rad 170–5,060) was used for detection, and Odyssey Fc imaging system (LI-COR Biosciences) and Image Studio (LI-COR Biosciences) were used for imaging.

Peak intensity of imaged bands were quantified in ImageJ and values were normalized to GAPDH (i.e., housekeeping gene). Normalized values were analyzed separately for males and female mice in R/R Studio using a two-way ANOVA, after confirming the requirements were met, to identify main effects of injury, genotype, and any interactions. Effect size was also calculated. Pairwise comparisons were subsequently performed via unpaired *t*-tests, comparing all experimental groups to WT sham, and each rmTBI group to its own respective sham. Statistical significance was determined as *p* < 0.05, and results were visualized using GraphPad Prism 8.0.1.

### Histology

Perfused and post-fixed brains in 4% paraformaldehyde were processed and embedded in formalin fixed paraffin blocks, with the right hemisphere (i.e., ipsilateral to impact) being used for staining. Blocks were cut along the sagittal plane at 5 um and sections were mounted onto slides.

*Immunofluorescence* was performed by the Pathology Research Program Laboratory associated with the University Health Network. Antigen retrieval was performed with low temperature citrate buffer (pH 6.0), and stained for BRCA1 (1:50, Abcam ab191042) followed by Alexa Fluor™ 555 secondary antibody for fluorescence (Invitrogen A21429). Representative images were taken at 40x magnification. For quantification, images were taken at 20x magnification on a confocal microscope and quantified in QuPath using positive cell detection to identify BRCA1-positive cells in the nuclear and cytoplasmic compartments. Positive cell detection was achieved using set intensity thresholds for DAPI fluorescence, followed by intensity threshold-based detection of nuclear and cytoplasmic BRCA1 fluorescence within detected cells. Five cortical images were taken from the same sagittal section, spanning the entire cortex (i.e., anterior to posterior), and averaged per sample (*n* = 3 per group). Results were analyzed using a two-way ANOVA to identify main effects of injury, cellular compartment, or interactions between these factors on the proportion of BRCA1-positive cells; effect size was also calculated. Pairwise comparisons were performed using unpaired *t*-tests between groups and visualized using GraphPad Prism 8.0.1. Statistical significance was determined as *p* < 0.05.

*Immunohistochemistry.* Slides were stained with hematoxylin and γH2AX (1:1,000, Abcam ab81299), followed by an anti-rabbit IMPRESS Reagent Kit Peroxidase (Vector Laboratories MP-7401) secondary antibody (*n* = 1). Images were scanned and viewed with QuPath.

### Dot blot

Nuclear fractionation and purification of genomic DNA via phenol chloroform extraction was performed on cortical tissue (*n* = 3 per group) from the right hemisphere (i.e., ipsilateral to impact). Double-stranded DNA (dsDNA) concentration was measured using the NanoDrop One UV–Vis Spectrophotometer (Thermofisher) followed by dilution in elution buffer to produce the following concentrations: 100 ng, 50 ng, 25 ng, 12.5 ng, and 6.25 ng. Samples were pipetted onto nitrocellulose membranes (Bio-Rad 1,620,112) and dried for 1 h. Membranes were blocked in 2% BSA in TBST for 2 h at room temperature. Membranes were incubated overnight in the following primary antibodies prepared in 1% BSA in TBST: anti-dsDNA for detection of DNA (1:20,000, Abcam ab27156), anti-S9.6 for detection of R-Loops (1:5,000, Sigma MABE1095), and anti-DNA/RNA oxidative damage for detection of 8-hydroxy-2′-deoxyguanosine, 8-hydroxyguanine, and 8-hydroxyguanosine (1:4,000, Abcam ab62623). Membranes were washed 3 times for 5 min in PBST, followed by a 2 h incubation at room temperature in HRP conjugated anti-mouse (1:10,000, Sigma-Aldrich A9044) secondary antibody prepared in 1% BSA in TBST. Membranes were washed for 15 min in PBST, followed by two washes for 15 min in PBS. Enhanced chemiluminescence substrate (Bio-Rad 170–5,060) was used for imaging on the Odyssey Fc imaging system (LI-COR Biosciences) with Image Studio (LI-COR Biosciences).

Intensity of dot blots were measured and quantified using ImageJ. Raw values were normalized to dsDNA for each sample. Normalized values were analyzed using a two-way ANOVA in R/RStudio to assess the effects of injury, genotype, or an interaction between injury and genotype in males and females separately; effect size was also calculated. Subsequent unpaired *t*-tests were performed for pairwise comparisons with statistical significance determined as *p* < 0.05. Results were visualized using GraphPad Prism 8.0.1.

## Results

### BRCA1 expression and subcellular localization in WT compared to HET mice 1-week following rmTBI

**Figure 1:**
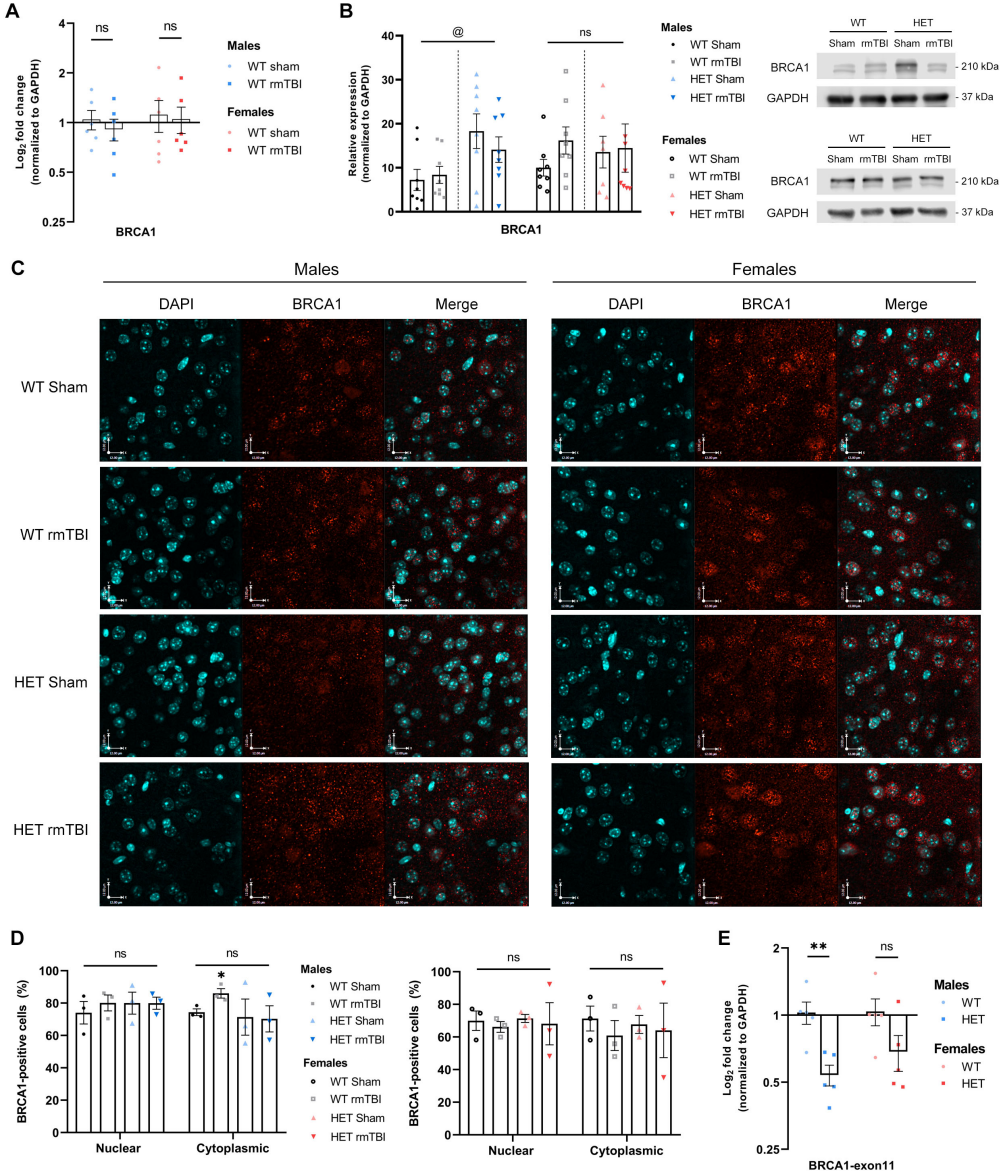
BRCA1 expression and subcellular localization in WT compared to HET mice 1-week following rmTBI. **(A)** Expression of total BRCA1 mRNA was measured using qPCR in WT mice and represented as mean log_2_ fold change ± SEM (*n* = 6 per group). Results were analyzed using unpaired *t*-tests (*). **(B)** BRCA1 protein expression was measured in WT and HET mice using Western blot (right) followed by quantification of band peak intensity (left), and is represented as mean relative expression ± SEM (*n* = 8 per group). Results were analyzed using two-way ANOVA to assess main effect of injury (#), genotype (@), or interaction (%). **(C,D)** Immunofluorescence was performed in males **(C, left)** and females **(C, right)** to assess BRCA1 localization in the nucleus and cytoplasm of WT and HET mice, followed by quantification **(D)** of BRCA1-positive cells in each compartment (mean BRCA1-positive cells ± SEM) (*n* = 3 per group). Representative images of the cortex, anterior to the hippocampus, are shown at 40x magnification and scale bars indicate 12.0 um. Results were analyzed using two-way ANOVA to assess effects of injury, compartment (&), or interaction on BRCA1 expression, followed by unpaired *t*-tests for pairwise comparisons (*). **(E)** Gene expression of wildtype exon 11 was measured using qPCR with a primer spanning exon 11 (*n* = 5 per group), proximal to the cassette insertion site in the mutant allele. Expression represented as mean log_2_ fold change ± SEM. Results were analyzed using unpaired *t*-tests. Statistical significance was determined as *p* < 0.05. Significance levels: *p* > 0.05 (ns), *p* < 0.05 (*, #, @, &, %), *p* < 0.01 (**, ##, @@, &&, %%), *p* < 0.001 (***, ###, @@@, &&&, %%%).

In previous research, BRCA1 was shown to be significantly downregulated in human brains with a history of repeated mTBI (Schwab et al., 2019), leading to deficient DNA repair and the onset of cellular senescence. In several neurodegenerative diseases, including Alzheimer’s disease and other tauopathies (Suberbielle et al., 2015; Kurihara et al., 2020), BRCA1 dysregulation and mislocalization have also been reported. As a primarily nuclear protein with crucial roles in maintaining genomic integrity, alterations in BRCA1 expression and localization may contribute to brain dysfunction. To validate this in mice, gene and protein expression of total BRCA1 was measured using qPCR and Western blot, respectively. 1-week following rmTBI, total BRCA1 expression in both WT males and females were not significantly changed and remained similar to WT sham counterparts (Figures 1A,B). When assessing BRCA1 localization using immunofluorescence to detect nuclear and cytoplasmic BRCA1-positive cells, a two way-ANOVA to evaluate effects of injury and subcellular compartment revealed no significant effects of either factor in males or females. However, with a pairwise comparison, males but not females with rmTBI exhibited a significant increase in cytoplasmic BRCA1-positive cells (*p* = 0.032, unpaired *t*-test), that was specific to WT mice, while maintaining similar levels of nuclear BRCA1-positive cells (Figures 1C,D). This suggests that translocation of BRCA1 post-injury in WT mice may impact BRCA1’s function in the DDR, and further impact outcomes following rmTBI.

To investigate the effects of reduced BRCA1-related repair, we evaluated DNA damage-induced senescence and outcomes following rmTBI in a mouse model with a heterozygous knockout for BRCA1, referred to as HET in future experiments. This heterozygous knockout for BRCA1 is achieved by insertion of a neocassette, disrupting intron 10 and exon 11 in the gene sequence to produce a null allele (Shen et al., 1998). Intact exon 11 is important to BRCA1’s function as it contains the nuclear localization signal needed for BRCA1 to operate as a nuclear DNA repair protein. Indeed, loss of exon 11 in BRCA1 results in impaired DDR (Huber et al., 2001; Wang et al., 2010). Validating this model using qPCR to measure wildtype exon 11 revealed a significant downregulation in wildtype exon 11 in males heterozygous for BRCA1 compared to WT mice (Figure 1E, *p* = 0.006, unpaired *t-*test), with a downregulation seen in females as well, although not significant (Figure 1E). To evaluate changes in BRCA1 expression in HET mice, BRCA1 protein levels were then measured in HET mice with and without rmTBI to compare how expression may differ to WT mice following rmTBI. When comparing BRCA1 protein levels between WT and HET mice, no significant effect of injury was detected using a two-way ANOVA. However, a significant effect of genotype (*p* = 0.014, η^2^ = 0.20, two-way ANOVA) was observed in males but not females, with HET shams expressing significantly higher levels of BRCA1 compared to WT sham counterparts (Figure 1B, *p* = 0.031, unpaired *t-*test). In contrast, BRCA1 levels in females were comparable between WT and HET mice (Figure 1B), suggesting that HET mice may exhibit some level of compensation for BRCA1. Further analysis of BRCA1 localization exhibited no overlying differences in nuclear or cytoplasmic BRCA1 expression due to injury or genotype in both males and females (Figures 1C,D). These results support that although HET mice overall exhibit reduced expression of wildtype exon 11, HET mice may exhibit compensation for BRCA1 expression to different degrees in males and females without additional mislocalization in this transgenic model. Next, we investigated both behavioural and molecular outcomes following rmTBI in WT and HET mice.

### Loss of righting reflex in WT and HET mice with rmTBI compared to sham

**Figure 2:**
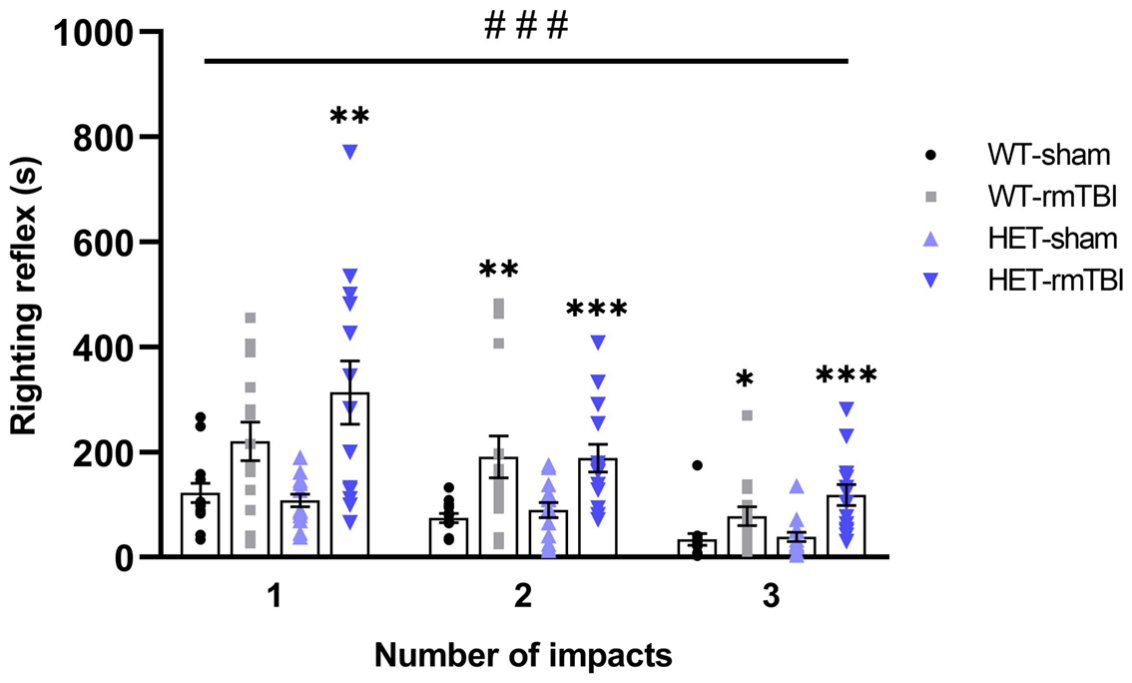
Loss of righting reflex in WT and HET mice with rmTBI compared to sham. Righting reflex was measured for each mouse following sham or rmTBI procedure and is represented as mean righting reflex ± SEM (*n* = 14 per group). Statistical significance was determined as *p* < 0.05 using a repeated measures two-way ANOVA to assess main effects of injury (#), genotype (@), or interaction (%), with unpaired *t-*test used for pairwise comparisons to WT shams (*). Significance levels: *p* < 0.05 (#, @, %, *), *p* < 0.01 (##, @@, %%, **), *p* < 0.001 (###, @@@, %%%, ***).

Righting reflex is often used as an indicator of injury, where a mild injury is attributed to a loss in righting reflex under 15 min (DeWitt et al., 2013). Thus, righting reflex was recorded and analyzed using a repeated measures two-way ANOVA following rmTBI and sham procedures in WT and HET mice (Figure 2). A significant effect of injury (*p* < 0.001, η^2^ = 0.37, repeated measures two-way ANOVA) was found, supporting a loss in righting reflex due to rmTBI. WT mice with rmTBI spent significantly more time to right following the second and third impact, while HET mice with rmTBI spent significantly more time to right following all impacts compared to their respective sham counterparts; *p*-values can be found in Table 1.

**Table 1:** *p*-values associated with the loss of righting reflex due to rmTBI 1-week following injury.

Day of impact	Comparison			
	WT-sham, WT-rmTBI	WT-sham, HET-sham	WT-sham, HET-rmTBI	HET-sham, HET-rmTBI
1	0.095	0.699	**0.002**	**0.001**
2	**0.008**	0.882	**<0.001**	**0.003**
3	**0.011**	0.465	**<0.001**	**<0.001**

Pairwise comparisons using unpaired *t*-tests were performed following a repeated measures two-way ANOVA. Statistical significance was determined as *p* < 0.05. Significant *p*-values are in bold.

### Decreased anxiety-related behaviours in males and heightened fear response in females with BRCA1 heterozygosity 1-week post-injury

**Figure 3:**
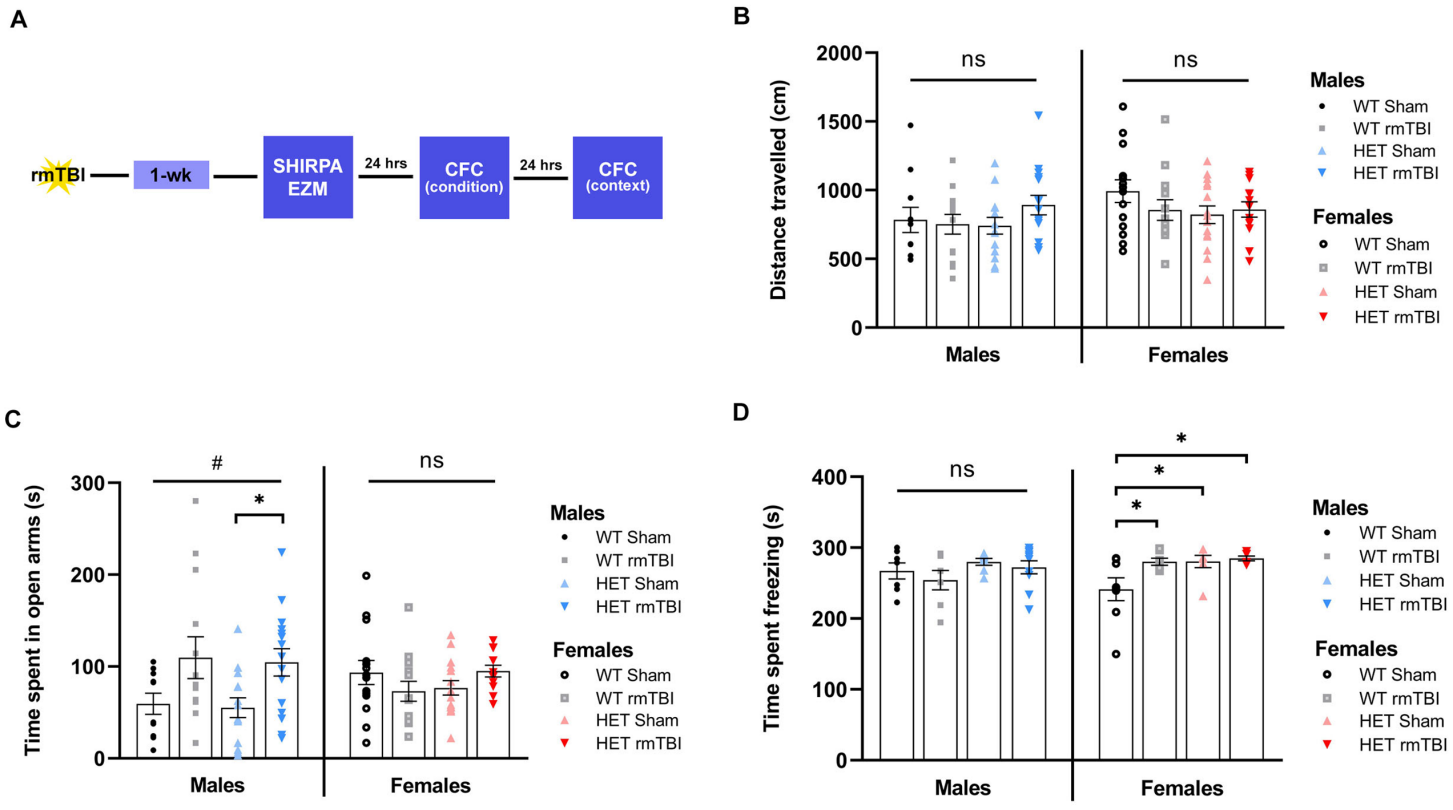
Decreased anxiety-related behaviours in males and heightened fear response in females with BRCA1 heterozygosity 1-week post-injury. **(A)** Schematic for behavioural paradigm used in this study 1-week post-rmTBI. **(B)** Distance travelled (mean ± SEM) and **(C)** time spent in open arms (mean ± SEM) was measured during elevated zero maze for males and females to examine exploratory behaviour and anxiety-related behaviour, respectively (*n* = 10–15 per group). **(D)** Time spent freezing (mean ± SEM) was measured during contextual fear conditioning to assess associative learning and memory (*n* = 6–10 per group). Statistical significance was determined as *p* < 0.05 using a two-way ANOVA to assess effects of injury (#), genotype (@), or interaction (%). Unpaired *t*-tests were performed for pairwise comparisons (*). Significance values: *p* > 0.05 (ns) *p* < 0.05 (#, @, %, *), *p* < 0.01 (##, @@, %%, **), *p* < 0.001 (###, @@@, %%%, ***).

1-week following rmTBI, mice underwent behavioural testing, including elevated zero maze (EZM) and contextual fear conditioning (CFC) to assess anxiety related behaviours and associative learning and memory, respectively (Figure 3A). Prior to behavioural testing, mice also underwent a phenotype assessment (i.e., SHIRPA), displaying similar physical appearance, reflexes, and mobility between WT and HET mice (Supplementary Figure S1).

Exploratory and anxiety-related behaviours were assessed using EZM (Tucker and McCabe, 2017) by measuring the total distance travelled and cumulative time spent in the open arms, respectively. A two-way ANOVA to evaluate effects of injury and genotype revealed no differences in the total distance travelled across all WT and HET groups (Figure 3B), suggesting comparable locomotor and exploratory activity among all groups. As for time spent in the open arms, a significant effect of injury was found (*p* = 0.011, η^2^ = 0.12, two-way ANOVA) in males but not females. In WT mice, males with rmTBI spent more time in the open arms compared to sham counterparts (Figure 3C, left). This effect was enhanced in HET males, where time spent in the open arms was significantly increased with rmTBI compared to shams (*p* = 0.015, unpaired *t-*test) (Figure 3C, left), suggesting decreased inhibition and anxiety-related behaviour following injury that is emphasized with BRCA1 heterozygous knockout. As for females, no significant effect of injury or genotype was found. A comparable amount of time was spent in the open arms for both WT and HET females with rmTBI compared to sham counterparts (Figure 3C, right), indicating no changes in anxiety-related behaviour measured by EZM.

Associative learning and memory were assessed using CFC (Maren et al., 2013) by measuring the amount of time spent freezing as an indicator of a learned fear response 24 h following fear conditioning in the same context. Using a two-way ANOVA to assess the effects of injury and genotype on time freezing, no significant main effects were found. At 1-week post-injury, WT males with rmTBI spent comparable time freezing to sham counterparts, with a similar effect observed for HET males with rmTBI (Figure 3D, left), exhibiting intact associative learning and memory. In contrast, WT females with rmTBI showed a significant increase in time spent freezing (*p* = 0.039, unpaired *t-*test) compared to sham counterparts (Figure 3D, right). Moreover, HET sham (*p* = 0.023, unpaired *t-*test) and rmTBI (*p* = 0.012, unpaired *t-*test) females spent significantly more time freezing compared to WT shams (Figure 3D, right), indicating an enhanced fear response in injured and HET females that was not seen in males.

### Elevated DNA damage in females and sex differences in DDR signalling due to BRCA1 heterozygosity 1-week post-rmTBI

**Figure 4:**
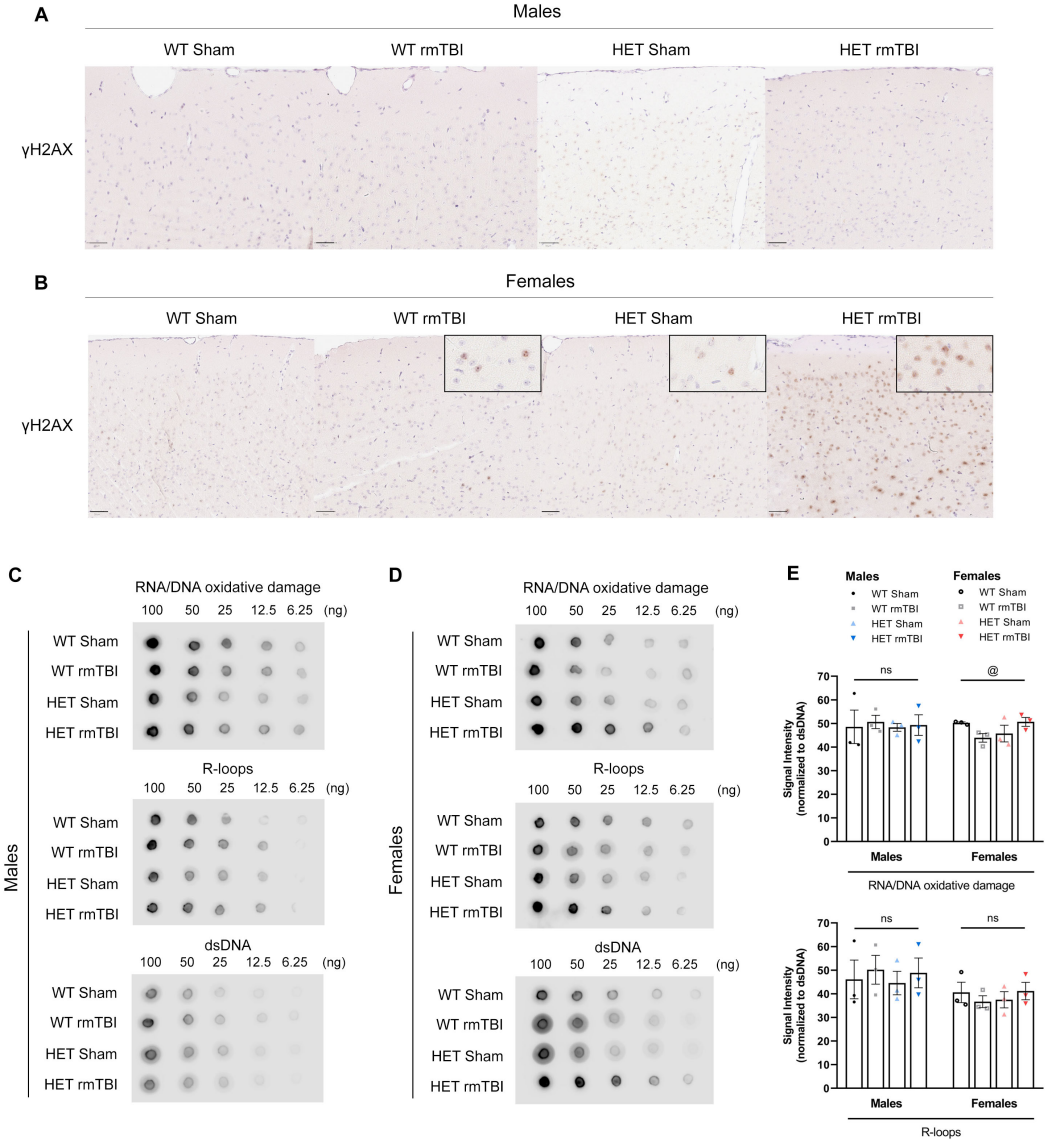
Elevated DNA damage in females due to BRCA1 heterozygosity 1-week post-rmTBI. DNA damage was assessed using immunohistochemistry **(A,B)** and dot blot **(C,E)**. Staining for γH2AX foci (brown) and hematoxylin (blue) were performed in males **(A)** and females **(B)** for all experimental groups (*n* = 1 per group) to assess double-stranded breaks. Representative images were taken from the cortex, anterior to the hippocampus. Scale bars represent 50 um. Dot blot was performed for R-loops and oxidative lesions at multiple dilutions (100 to 6.25 ng) relative to dsDNA to measure additional sources of DNA damage in males **(C,E)** and females **(D,E)** for all experimental groups (*n* = 3 per group). Statistical significance was determined as *p* < 0.05 using a two-way ANOVA to assess effects of injury (#), genotype (@), or interaction (%). Significance levels: *p* > 0.05 (ns), *p* < 0.05 (#, @, %), *p* < 0.01 (##, @@, %%), *p* < 0.001 (###, @@@, %%%).

With evident DNA damage present following injury (Schwab et al., 2019; Davis and Vemuganti, 2021), changes in DNA damage and DDR signalling as a result of deficient DNA repair were investigated. γH2AX foci, a prominent marker of double-stranded breaks (DSBs) (Mah et al., 2010; Rahmanian et al., 2021), was evaluated using immunohistochemistry following rmTBI in WT and HET mice. In males, minimal changes were observed in γH2AX foci with rmTBI in both WT and HET mice (Figure 4A). However in females, an increase in γH2AX foci were seen in WT rmTBI mice compared to shams that was further increased in HET females with rmTBI (Figure 4B). Additional sources of DNA damage that BRCA1 is involved in repairing, including RNA/DNA oxidative lesions (Le Page et al., 2000) and R-loops (Hatchi et al., 2015) were also detected using dot blot in both males and females. In males, a two-way ANOVA revealed no significant effects of injury, genotype or interaction on levels of R-loops and oxidative lesions (Figures 4C,E), with similar expression observed between experimental groups. However in females, although no significant effects were seen in the expression of R-loops, a significant interaction between injury and genotype was identified for oxidative lesions (*p* = 0.032, η^2^ = 0.13, two-way ANOVA) (Figures 4D,E). Oxidative lesions were seen to decrease in WT females with rmTBI, but increase in HET females with rmTBI compared to respective sham counterparts, however no significant pairwise comparisons were found. These results suggest that although levels of DNA damage may not exhibit notable changes in WT male or female mice, BRCA1 heterozygosity may promote elevated DNA damage in the form of DSBs and oxidative lesions in females but not males with rmTBI.

**Figure 5:**
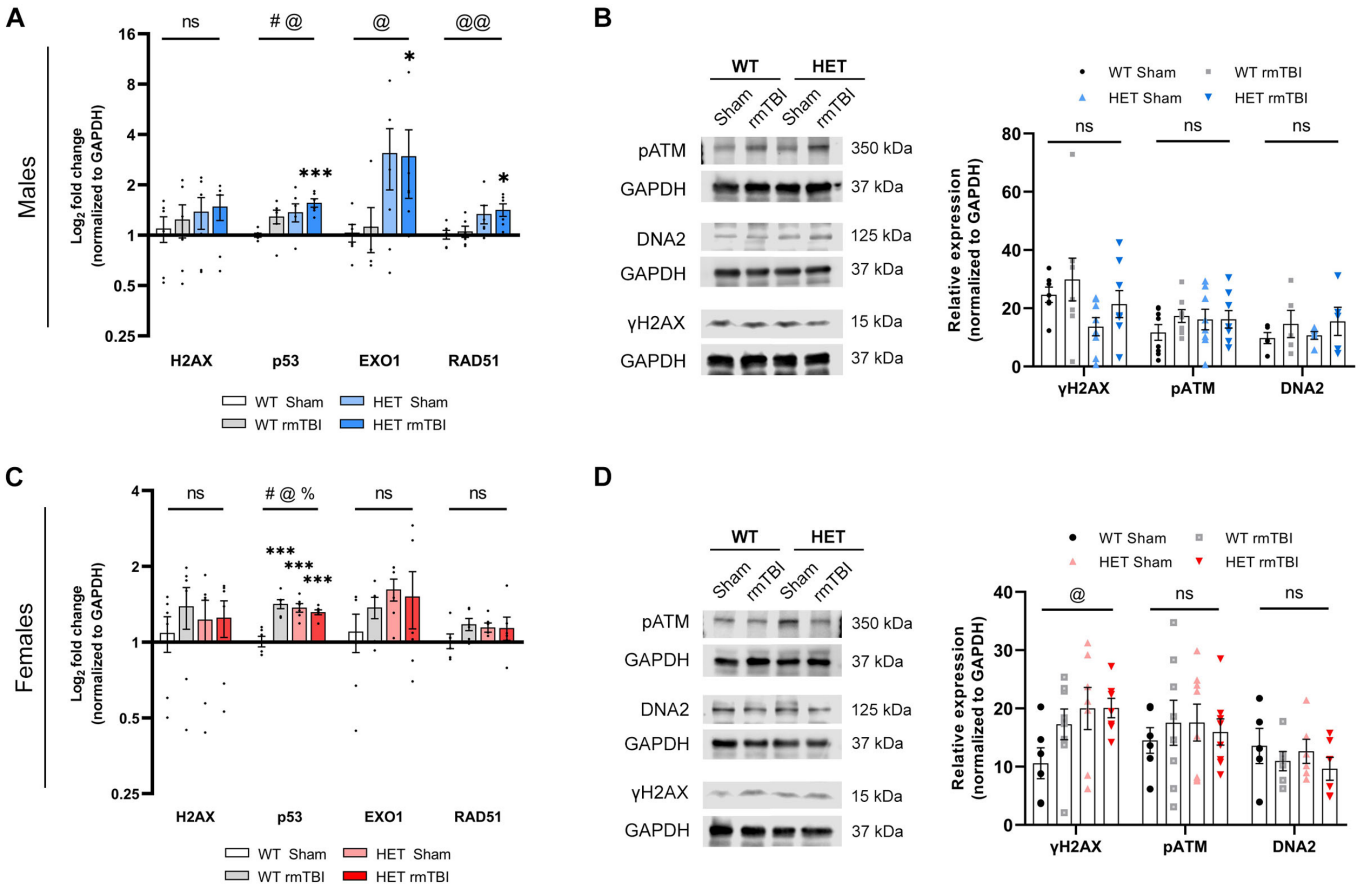
Sex differences in DDR signalling due to BRCA1 heterozygosity 1-week post-rmTBI. Activation of the DDR was assessed using qPCR (*n* = 6 per group) and Western blots (*n* = 5–8 per group) in males **(A,B)** and females **(C,D)** 1-week post-injury. H2AX, p53, EXO1 and RAD51 mRNA expression were measured with qPCR and represented as log_2_ fold change (mean ± SEM) **(A,C)**. Protein expression of yH2AX, pATM, and DNA2 were measured using Western blot **(B,D; left)**, followed by quantification of peak band intensity and represented as relative expression (mean ± SEM) **(B,D; right)**. Statistical significance was determined as *p* < 0.05 using two-way ANOVA to assess main effect of injury (#), genotype (@), or interaction (%), with unpaired *t*-test used for pairwise comparisons to WT shams (*). Significance levels: *p* > 0.05 (ns), *p* < 0.05 (#, @, %, *), *p* < 0.01 (##, @@, %%, **), *p* < 0.001 (###, @@@, %%%, ***).

Expression of several targets involved in the DDR were measured using qPCR and Western blot, and analyzed using a two-way ANOVA to assess significant effects of injury and/or genotype separately in males and females 1-week post-injury (Figure 5).

In males, a significant effect of injury (*p* = 0.043, η^2^ = 0.19, two-way ANOVA) and genotype (*p* = 0.016, η^2^ = 0.26, two-way ANOVA) on p53 (Lakin and Jackson, 1999) gene expression was observed, with WT rmTBI (*p* = 0.069, unpaired *t-*test) and HET shams (*p* = 0.065, unpaired *t-*test) showing an increase in expression, and HET rmTBI males showing a significant increase in p53 expression (*p* < 0.001, unpaired *t-*test) compared to WT sham counterparts (Figure 5A). Although no significant changes were seen for H2AX, EXO1 (Bolderson et al., 2010) (*p* = 0.017, η^2^ = 0,25, two-way ANOVA) and RAD51 (Bonilla et al., 2020) (*p* = 0.005, η^2^ = 0.33, two-way ANOVA) gene expression were significantly affected by genotype (Figure 5A), where HET males display greater expression compared to WT males. Specifically, HET males with rmTBI had significantly greater expression of EXO1 (*p* = 0.047, unpaired *t-*test) and RAD51 (*p* = 0.012, unpaired *t-*test) compared to WT shams (Figure 5A), suggesting an upregulation in DNA repair genes not seen in WT males with rmTBI. Protein expression of γH2AX and additional DNA damage sensing and repair proteins pATM (Maréchal and Zou, 2013) and DNA2 (Pawłowska et al., 2017), respectively, were not significantly affected by injury or genotype (Figure 5B). However, γH2AX and DNA2 expression were seen to increase following rmTBI in both WT and HET mice, while pATM expression was increased in all experimental groups compared to WT shams, although these results were not significant (Figure 5B).

**Figure 6:**
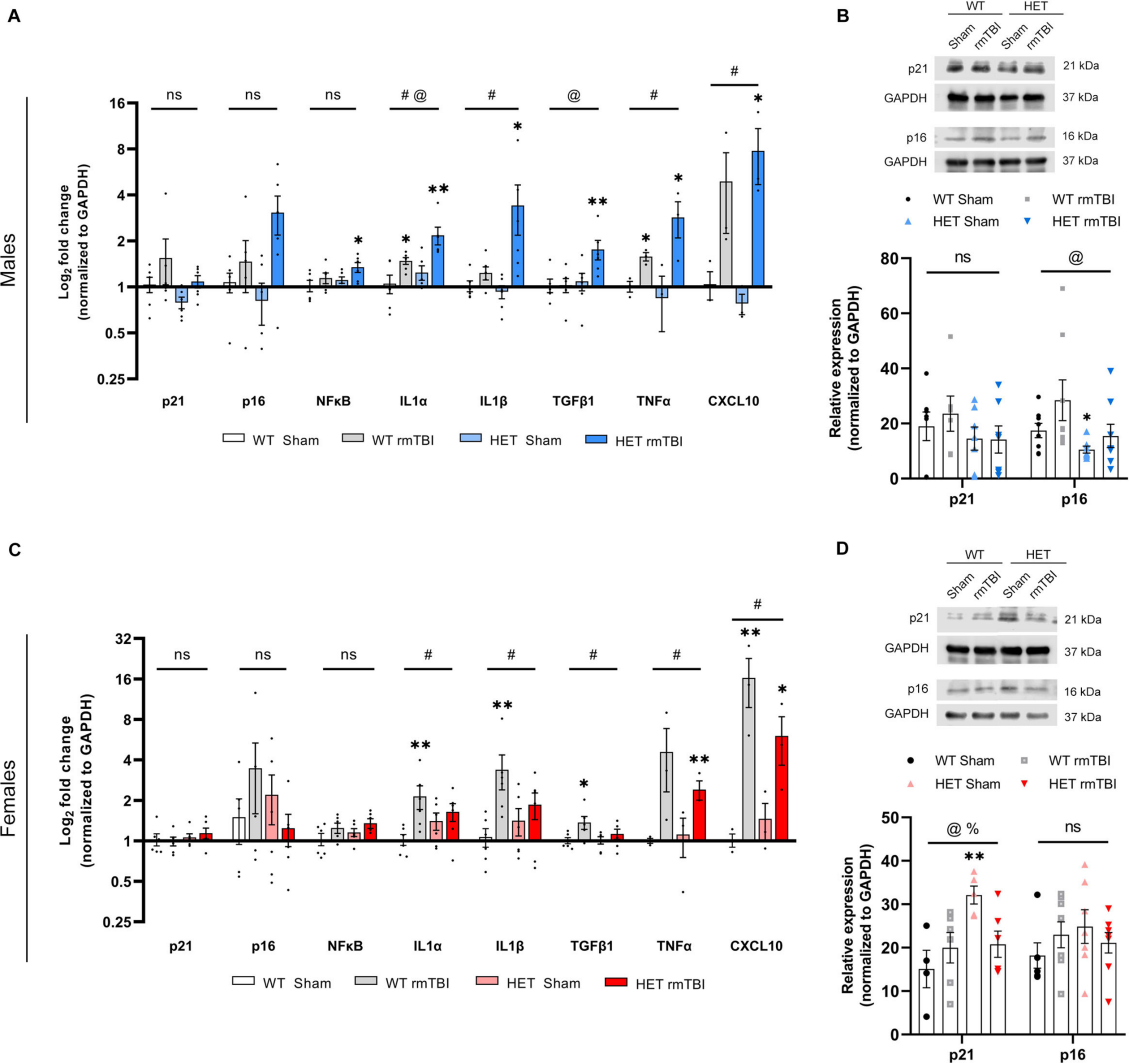
Robust senescence in males and diminished senescence response in females due to BRCA1 heterozygosity 1-week post-rmTBI. Activation of senescence-associated pathways and SASP-related factors were assessed for males and females 1-week post-injury. Gene expression was measured using qPCR (*n* = 3–6 per group) for males **(A)** and females **(C)**, represented as a log_2_ fold change (mean ± SEM). Protein expression of p21 and p16 were also validated using Western blots (*n* = 5–8 per group) in males **(B, top)** and females **(D, top)**, with quantification of peak intensity bands represented as relative expression (mean ± SEM) for males **(B, bottom)** and females **(D, bottom)**. Statistical significance was determined as p < 0.05 using a two-way ANOVA to assess main effects of injury (#), genotype (@), or interaction (%), followed by unpaired *t-*tests for pairwise comparisons between experimental groups to WT shams (*).: Significance levels: *p* > 0.05 (ns), *p* < 0.05 (#, @, %, *), *p* < 0.01 (##, @@, %%, **), *p* < 0.001 (###, @@@, %%%, ***).

In females, p53 gene expression was also significantly affected by injury (*p* < 0.001, η^2^ = 0.43, two-way ANOVA) and genotype (*p* = 0.006, η^2^ = 0.32, two-way ANOVA), with a significant interaction between injury and genotype observed as well (*p* < 0.001, η^2^ = 0.55, two-way ANOVA) (Figure 5C). Significant upregulation of p53 was seen in WT rmTBI (*p* > 0.001, unpaired *t-*test), HET sham (*p* < 0.001, unpaired *t-*test), and HET rmTBI (*p* < 0.001, unpaired *t-*test) mice compared to WT sham counterparts (Figure 5C). In contrast to males, gene expression of H2AX, EXO1 and RAD51 were comparable across experimental groups, exhibiting no significant effect of injury or genotype (Figure 5C). However, at the protein level, a significant effect of genotype (*p* = 0.043, η^2^ = 0.16, two-way ANOVA) was observed for γH2AX expression, with increased γH2AX expression in HET sham and HET rmTBI mice compared to WT shams, further supporting elevated DNA damage burden in BRCA1 heterozygous knockout mice. No significant effect of injury or genotype was seen for pATM and DNA2 expression (Figure 6D).

Overall, these results suggest initial activation of the DDR in both WT males and females, with upregulation of p53 expression, and markers of DNA damage and repair following injury. HET mice also exhibited initial DDR signalling following rmTBI, with HET female mice displaying elevated DNA damage burden compared to WT sham mice.

### Robust senescence in males and diminished senescence response in females due to BRCA1 heterozygosity 1-week post-rmTBI

Previously reported in the literature, cellular senescence can be induced by genotoxic stress (d’Adda di Fagagna, 2008; Schwab et al., 2019, 2021). Once established, senescence is often accompanied by a senescence-associated secretory phenotype (SASP), involving the increased production and secretion of a myriad of pro-inflammatory factors (Salminen et al., 2011; Cheng et al., 2017). Thus, to evaluate the onset of cellular senescence, markers involved in senescence induction, maintenance, and SASP were evaluated in this study using qPCR and Western blot.

Male mice exhibited a trending effect of injury on p21 (*p* = 0.074, two-way ANOVA), a cell cycle regulator associated in senescence induction (Stein et al., 1999; He and Sharpless, 2017), p16 (*p* = 0.088, two-way ANOVA), involved in senescence maintenance (Stein et al., 1999; He and Sharpless, 2017), and NFκB (*p* = 0.052, two-way ANOVA), involved in senescence-associated processes and activated by DNA damage (Salminen et al., 2012; Tilstra et al., 2012), although these results were not significant (Figure 6A). However, p21 and p16 were significantly increased in HET rmTBI mice compared to HET sham counterparts, while NFκB was significantly upregulated in HET mice with rmTBI compared to WT shams (*p*-values can be found in Table 2). At the protein level, p16 expression was significantly affected by genotype but not injury (*p* = 0.040, η^2^ = 0.15, two-way ANOVA), and was significantly decreased (*p* = 0.040, unpaired *t-*test) in HET shams compared to WT sham counterparts (Figure 6B). As for targets associated with the SASP, gene expression of several targets were significantly impacted by injury and genotype. Using a two-way ANOVA, a significant effect of injury was found for IL1α (*p* < 0.001, η^2^ = 0.49), IL1β (*p* = 0.002, η^2^ = 0.38), TNFα (*p* = 0.018, η^2^ = 0.52), and CXCL10 (*p* < 0.001, η^2^ = 0.77), whereas genotype had a significant effect on IL1α (*p* = 0.019, η^2^ = 0.25) and TFGβ1 expression (*p* = 0.035, η^2^ = 0.20) (Figure 6A). In support of this, WT mice showed a significant upregulation in IL1α and TNFα expression post-rmTBI compared to WT shams, whereas HET mice showed significant upregulation of IL1α, IL1β, TGFβ1, and TNFα post-rmTBI compared to WT and HET sham counterparts (Figure 6A, *p*-values can be found in Table 2). These results indicate activation of senescence-associated pathways in WT mice following rmTBI that may be exacerbated with BRCA1 heterozygous knockout with a more severe SASP expression.

**Table 2:** *p*-values associated with changes in expression of senescence- and SASP-related genes 1-week post-rmTBI in WT and HET male mice.

Target	Comparison			
	WT-sham, WT-rmTBI	WT-sham, HET-sham	WT-sham, HET-rmTBI	HET-sham, HET-rmTBI
p21	0.373	0.126	0.715	**0.036**
p16	0.917	0.269	0.060	**0.042**
NFκB	0.330	0.337	**0.034**	0.067
IL1α	**0.027**	0.298	**0.002**	**0.005**
IL1β	0.151	0.460	**0.014**	**0.010**
TGFβ1	0.981	0.844	**0.009**	**0.033**
TNFα	**0.012**	0.496	**0.036**	0.088
CXCL10	0.074	0.327	**0.010**	**0.005**

Pairwise comparisons using unpaired *t*-tests were performed following a two-way ANOVA. Statistical significance was determined as *p* < 0.05. Significant *p*-values are in bold.

In contrast, females did not display a significant effect of injury or genotype on p21, p16, or NFkB gene expression (Figure 6C). However, a significant effect of genotype on p21 protein expression (*p* = 0.031, η^2^ = 0.25, two-way ANOVA) was observed, with a significant effect of interaction between injury and genotype (*p* = 0.025, η^2^ = 0.26, two-way ANOVA) as well (Figure 6D). Protein expression of p21 was significantly upregulated in HET shams compared to WT shams (*p* = 0.007, unpaired *t-*test), and significantly downregulated in HET rmTBI mice (*p* = 0.016, unpaired *t-*test) compared to HET sham counterparts, suggesting differential activation of senescence-associated pathways in WT and HET females with rmTBI. With regards to SASP-related factors, a two-way ANOVA revealed only a significant effect of injury in females for IL1α (*p* = 0.017, η^2^ = 0.25), IL1β (*p* = 0.011, η^2^ = 0.28), TFGβ1 (*p* = 0.025, η^2^ = 0.23), TNFα (*p* = 0.015, η^2^ = 0.54), and CXCL10 (*p* < 0.001, η^2^ = 0.80) (Figure 6C). Accordingly, IL1α, IL1β, TGFβ1, and CXCL10 were significantly upregulated in WT rmTBI mice, and CXCL10 and TNFα were significantly upregulated in HET rmTBI mice compared to WT shams (Figure 6C, *p*-values can be found in Table 3).

**Table 3:** *p*-values associated with changes in expression of senescence- and SASP-related genes 1-week post-rmTBI in WT and HET female mice.

Target	Comparison			
	WT-sham, WT-rmTBI	WT-sham, HET-sham	WT-sham, HET-rmTBI	HET-sham, HET-rmTBI
p21	0.871	0.660	0.426	0.602
p16	0.281	0.563	0.918	0.548
NFκB	0.182	0.322	0.065	0.200
IL1α	**0.009**	0.139	0.060	0.556
IL1β	**0.005**	0.429	0.195	0.538
TGFβ1	**0.030**	0.982	0.357	0.403
TNFα	0.078	0.926	**0.006**	0.117
CXCL10	**0.005**	0.402	**0.021**	0.060

Pairwise comparisons using unpaired *t*-tests were performed following a two-way ANOVA. Statistical significance was determined as *p* < 0.05. Significant *p*-values are in bold.

Together these results suggest that although WT mice exhibit activation of senescence-associated pathways with rmTBI, HET mice display opposing phenotypes between males and females with rmTBI, revealing potential sex-specific differences associated with BRCA1 heterozygous knockout. More specifically, despite an elevated DNA damage burden previously observed in HET females, senescence-associated pathways are promoted in males but not females with BRCA1 heterozygosity.

## Discussion

The objective of this study was to evaluate the consequences of DNA repair deficiency on DNA damage-induced senescence and behavioural outcomes following rmTBI. Here we used a controlled cortical impact model of rmTBI in mice with a heterozygous knockout for BRCA1 to investigate the effects of impaired BRCA1-related repair on outcomes 1-week following injury. Given the evident sex-specific differences in outcomes and recovery associated with mTBI in humans (Bazarian et al., 2010; Villapol et al., 2017; Gupte et al., 2019), sex differences were also addressed in this study by including sex-balanced experimental groups.

In this study, we showed a significant increase in cytoplasmic BRCA1-positive cells with rmTBI specifically in WT males. Although BRCA1 has functions in the cytoplasm, including maintaining centrosome function in dividing cells and regulating apoptotic signalling (Deng and Brodie, 2000; Fabbro et al., 2004; Jiang et al., 2011), its dysregulation in various neurodegenerative diseases (Noristani et al., 2015; Suberbielle et al., 2015; Kurihara et al., 2020) where BRCA1 is translocated to the cytoplasm suggests that excessive mislocalization of BRCA1 may contribute to brain dysfunction. Despite the potential dysregulation of BRCA1 observed in males with rmTBI, the proportion of nuclear BRCA1-positive cells remained unchanged with rmTBI in both males and females. Autoregulation of BRCA1 has been reported in cells, where BRCA1-deficient cells have been shown to increase BRCA1 transcription in the presence of genotoxic stress (De Siervi et al., 2010). Due to increased genomic instability and genotoxic stress following injury, a similar autoregulatory mechanism may explain the maintenance of BRCA1 expression in the nucleus.

Interestingly, HET mice did not exhibit a downregulation in BRCA1 expression or subcellular mislocalization. Instead, BRCA1 protein levels were comparable to that of WT in females, while HET males expressed significantly more BRCA1 compared to WT counterparts, further supporting the possibility of a compensatory mechanism. However, despite similar levels of BRCA1 seen in HET compared to WT mice, HET mice with rmTBI exhibited different outcomes from WT mice in a sex-specific manner. This suggests that although BRCA1 levels and localization in the brain appear sufficient in HET mice, there may be altered function, other affected pathways, or regulatory mechanisms due to the heterozygous knockout promoting differential outcomes post-injury in males and females. For instance, different brain cell types may exhibit differential BRCA1 regulation. Indeed, in response to DSBs induced by stabilized G-quadraplex DNA structures, a downregulation of BRCA1 was shown in neurons, but not microglia or astrocytes (Tabor et al., 2021). In this study, measuring BRCA1 levels was done in a heterogeneous population of brain cell types, suggesting that cell-specific regulation of BRCA1 may also occur post-injury that could not be discerned in this study.

Common symptoms experienced by individuals following rmTBI include alterations in anxiety and depression-like symptoms, as well as reduced cognitive function (Dean and Sterr, 2013; Wickwire et al., 2018). Accordingly, we assessed these changes in this study using EZM and CFC. At 1-week post-rmTBI, behavioural changes were found in BRCA1 heterozygous knockout animals, with distinct behavioural changes observed in males versus females. HET males with rmTBI exhibited intact associative learning and memory with CFC, but displayed increased risk-taking in the EZM. In contrast, HET females with rmTBI exhibited minimal changes to risk-taking behaviour measured by EZM, but displayed an enhanced fear response with CFC. Although alterations to cognition were not observed at 1-week post-injury in males or females, it could be speculated that both exhibited changes in anxiety-related behaviour. For instance, CFC is commonly used in studies focused on post-traumatic stress disorder (Bienvenu et al., 2021), where a heightened fear response may act as an alternative indicator of increased stress and anxiety. This may suggest that changes in anxiety occur in both sexes but are captured differently based on sex. In support of this, a recent study investigating the effects of deficient DNA repair on multi-symptomatic disorders found that mice with a conditional knockout for XRCC1 (i.e., a BER protein) displayed increased anxiety in males using the light–dark box test, while increased fear learning was observed in females using fear conditioning (Mueller et al., 2022). Thus, altered DNA repair, elicited here by a BRCA1 heterozygous knockout, may work in a similar manner to differentially alter anxiety-related behaviours in both males and females with rmTBI.

In addition to identifying sex differences in behavioural outcomes, an important aim for this study was to evaluate the consequences of aberrant DNA repair in DNA damage-induced senescence, and associated sex-specific outcomes, as a driver of brain dysfunction. In line with previous studies (Schwab et al., 2021), both male and female WT mice showed activation of the DDR and senescence-associated pathways, with upregulation of p53, DNA repair proteins, and multiple SASP-related factors. With addition of the BRCA1 heterozygous knockout, molecular changes were seen even without injury, and divergent molecular phenotypes were identified following rmTBI. Significant upregulation of p21, p16, and NFkB gene expression were seen in HET males with rmTBI compared to sham counterparts with a significant effect of both injury and genotype on various SASP-related factors. In contrast, females exhibited a significant interaction between injury and genotype on p21 expression, where p21 was significantly decreased following rmTBI in HET mice, and only a significant effect of injury was found for SASP-related factors. Therefore, these results support a less additive effect of injury and genotype in females compared to males in the activation of senescence-associated pathways. These results suggest that while males exhibited a robust DDR and senescence response following rmTBI, females exhibited a diminished response despite an elevated DNA damage burden. Moreover, expression of DNA repair proteins also displayed opposing trends based on sex, where EXO1, RAD51 and DNA2—proteins that interact with BRCA1 in DNA repair—were upregulated in HET males, with minimal changes seen in HET females with rmTBI. This novel finding further reinforces sex differences in the DDR, and provides evidence that these differences may be BRCA1-dependent. Sufficient BRCA1 compensation and increased DNA repair may reinforce proper signalling of the DDR and downstream senescence-associated pathways in HET males post-rmTBI, whereas lack thereof in HET females may result in impaired DDR signalling, leading to a dampened senescence response with greater DNA damage.

Looking at DNA repair more broadly, disparities between sexes have been identified for both single and double-stranded DNA repair pathways. For instance, DNA repair capacity of DSBs measured as the frequency of HR, NHEJ, and microhomology-mediated end joining (MMEJ), was shown to decline with age in women compared to men (Rall-Scharpf et al., 2021). In addition to changes in DNA repair function, shifts in repair pathways can also occur if a preferred pathway is unavailable. Indeed, when HR proteins, such as BRCA1, were depleted in human cells, MMEJ was upregulated, promoting more error-prone repair, genotoxic stress, and chromosomal rearrangements (Ahrabi et al., 2016). Whether male and female mice with rmTBI in this study underwent shifts in DNA repair capacity as a result of BRCA1 heterozygosity was not explored in this study and would require further investigation.

Due to sex differences in DDR signalling in BRCA1 HET mice, males and females exhibited contrasting activation of downstream senescence pathways. While HET males with rmTBI exhibited upregulation in senescence-associated markers and various SASP-related factors, HET females with rmTBI exhibited a downregulation in senescence-associated markers with moderate upregulation of SASP-related factors. With more specificity towards BRCA1’s role in senescence, BRCA1 has been reported to both promote and prevent senescence. BRCA1 deficiency has been associated with p16 induction, thereby promoting premature senescence (Scott et al., 2017), but has also been associated with reduced p21 expression to promote proliferation (Somasundaram et al., 1997; Zeng et al., 2020). Thus, the role of BRCA1 in senescence may be context-dependent, and activate alternative pathways between males and females, where BRCA1 heterozygous knockout leads to a more senescent phenotype in males and a more proliferative phenotype in females, although further studies are needed for validation.

Overall, although these findings are limited in their translatability, the emphasis on sex-specific outcomes and potential treatments is evident. Concluding that different molecular outcomes are present between males and females with altered BRCA1-related repair suggests that treatment methods in males may not have the same efficacy in females. For instance, the use of senolytics to remove senescent cells has shown success in males with adverse effects in females. Following treatment of a senolytic cocktail in C57BL/6 mice, male mice showed reduced SASP and improved cognitive performance using Morris water maze, while female mice showed exacerbated SASP and reduced cognitive performance using novel object recognition task (Fang et al., 2022). Similar outcomes were also recently reported in C57BL/6 mice with rmTBI, where a reduction in senescence-related markers and improved spatial learning and memory were seen in treated males, but not females (Schwab et al., 2022). Therefore, alternative therapeutic strategies should be explored for treating females versus males. Potential therapeutic avenues focused on boosting DNA repair capacity for instance may be more beneficial for females. Indeed, treatments directed at upregulating DNA repair capacity have been reported in the literature, where cells treated with small non-coding RNAs that help facilitate the DDR were more resilient to genotoxic insults from ionizing radiation compared to vehicle-treated cells (Gioia et al., 2019). Low-dose radiation (LDR) is another method that has been shown to promote DNA repair, reduce DNA damage, and attenuate oxidative stress (Xu et al., 2022). In the context of injury, LDR treatment prior to doxorubicin-induced brain injury in BALB/C mice was shown to combat oxidative damage and downregulate expression of proapoptotic proteins (Gao et al., 2022). Cumulatively these studies not only emphasize the importance of sufficient DNA repair to mitigate consequences of stress and insults, but also the need to explore alternative therapeutic avenues to effectively treat symptoms following rmTBI in a sex-specific manner.

## Conclusion

As mTBI remains a growing public health issue, research into underlying mechanisms driving sequelae associated with injury is imperative to the development of effective treatment strategies. With emerging evidence supporting elevated DNA damage with insufficient DNA repair in the context of TBI, this study evaluated the effects of aberrant BRCA1-related repair on DNA damage-induced senescence and outcomes following rmTBI in mice with a heterozygous knockout for BRCA1. While WT mice with rmTBI exhibited minimal changes in behaviour, mislocalization of BRCA1, and activation of the DDR and senescence-associated pathways, HET mice with rmTBI exhibited changes in anxiety-related behaviours and distinct molecular phenotypes between males and females. More specifically, HET rmTBI males displayed a robust senescence response whereas HET rmTBI females displayed impaired DDR signalling followed by a diminished senescence response, suggesting sex differences in the DDR that may be BRCA1-dependent. Therefore, this study not only highlights the importance of proper DNA repair following rmTBI, but also the need to develop therapeutic strategies in a sex-specific manner.

## Data availability statement

The original contributions presented in the study are included in the article/Supplementary material, further inquiries can be directed to the corresponding author.

## Ethics statement

The animal study was reviewed and approved by the Centre of Phenogenomics Animal Care Committee.

## Author contributions

EL: animal experiments, collection of data, data analysis including statistical tests, manuscript writing; DT: collection of dot blot data and qPCR data, manuscript input; NS: collection of dot blot data, manuscript input; LNH: study design, funding acquisition, manuscript input, supervision. All authors contributed to the article and approved the submitted version.

## Funding

This research was funded by the Canadian Institutes of Health Research (CIHR); Grant# 6210100803.

## Conflict of interest

The authors declare that the research was conducted in the absence of any commercial or financial relationships that could be construed as a potential conflict of interest.

## Publisher’s note

All claims expressed in this article are solely those of the authors and do not necessarily represent those of their affiliated organizations, or those of the publisher, the editors and the reviewers. Any product that may be evaluated in this article, or claim that may be made by its manufacturer, is not guaranteed or endorsed by the publisher.

## Abbreviations

BRCA1: breast cancer type I
CFC: contextual fear conditioning
DDR: DNA damage response
DSB: double-stranded break
dsDNA: double-stranded DNA
EZM: elevated zero maze
HET: heterozygous knockout for BRCA1
HR: homologous recombination
LDR: low-dose radiation
MMEJ: microhomology-mediated end joining
mTBI: mild traumatic brain injury
NHEJ: non-homologous end joining
rmTBI: repeated mild traumatic brain injury
SASP: senescence-associated secretory phenotype
WT: wildtype
