# Supplementary material for: BRCA1 heterozygosity promotes DNA damage-induced senescence in a sex-specific manner following repeated mild traumatic brain injury
Source: Front Neurosci. 2023 Aug 10;17:1225226. doi: 10.3389/fnins.2023.1225226 (PMC10450634; doi:10.3389/fnins.2023.1225226)
Supplement: Supplementary file 1 [file Data_Sheet_1.docx]

**Supplementary Table 1.** List of gene targets including forward (F) and reverse (R) primer sequences used for qPCR. Housekeeping gene is in bold.

| **Target** | **F/R** | **Sequence** |
| --- | --- | --- |
| H2AX | F | CATACCAGTTGACCCTGTCG |
|  | R | GGTAAGCGTCTCTCTGACCC |
| p53 | F | AGCTTTGAGGTTCGTGTTTGTG |
|  | R | TGGGCAGCGCTCTCTTTG |
| EXO1 | F | TGTGTGCTATCAGGGTCAGT |
|  | R | TGCAGTGCCTCCTTGTGTTA |
| RAD51 | F | ATCCCTGCATGCTTGTTCTC |
|  | R | CTGCAGCTGACCATAACGAA |
| IL1a | F | CAAACTGATGAAGCTCGTCA |
|  | R | TCTCCTTGAGCGCTCACGAA |
| IL1b | F | CCAGGATGAGGACATGAGCA |
|  | R | TCATCTCGGAGCCTGTAGTG |
| TGFB1 | F | CTGGGCACCATCCATGACA |
|  | R | GCCGCACACAGCAGTTCTT |
| p21 (CDKN1A) | F | TGTCGCTGTCTTGCACTCTG |
|  | R | GACCAATCTGCGCTTGGAGT |
| p16 (CDKN2A) | F | CCGAACTCTTTCGGTCGTAC |
|  | R | AGTTCGAATCTGCACCGTAGT |
| NFkB (RelA/p65) | F | GGAACAGGTGGGATGTTGCT |
|  | R | GACTAAACTCCCCCTGATTCTGAAG |
| TNFa | F | GGTGCCTATGTCTCAGCCTCTT |
|  | R | GCCATAGAACTGATGAGAGGGAG |
| CXCL10 | F | ATCATCCCTGCGAGCCTATCCT |
|  | R | GACCTTTTTTGGCTAAACGCTTTC |
| BRCA1 | F | TTGCCTGCCAAGGCGAGA |
|  | R | TGCCAATGTGGGCTGGCT |
| BRCA1-ex11 | F | CAAACAGCCTGGCATAGCAG |
|  | R | GCTGCTATTTAGTGTTATCCAAGG |
| **GAPDH** | F | CCAATGTGTCCGTCGTGGATCT |
|  | R | GTTGAAGTCGCAGGAGACAACC |

**Supplementary Figure 1. Comparable weight, length, and locomotor activity between WT and HET male and female mice 1-week following rmTBI.** A phenotype assessment was completed prior to behavioural testing where weight (A), length (B), and locomotor activity (C) was measured and recorded to identify notable physical differences. Length was measured from nose to base of tail, while locomotor activity was measured by counting the number of squares within a gridded arena crossed in a 30 s time period. Statistical significance was determined as p<0.05 using a two-way ANOVA to assess main effects of injury (#), genotype (@), or interaction (%) on the aforementioned metrics in males and females. No significant differences were found for males and females (denoted as ns).
